# Celecoxib regulates apoptosis and autophagy via the PI3K/Akt signaling pathway in SGC-7901 gastric cancer cells

**DOI:** 10.3892/ijmm.2014.1713

**Published:** 2014-03-27

**Authors:** MIN LIU, CHUN-MEI LI, ZHAO-FENG CHEN, RI JI, QING-HONG GUO, QIANG LI, HONG-LING ZHANG, YONG-NING ZHOU

**Affiliations:** 1 Division of Gastroenterology and Hepatology, The First Hospital of Lanzhou University, Lanzhou, Gansu 730000, P.R. China; 2 Division of Oncology, The First Hospital of Lanzhou University, Lanzhou, Gansu 730000, P.R. China

**Keywords:** apoptosis, autophagy, celecoxib, cyclooxygenase 2, Akt, caspase-8, caspase-9, gastric carcinoma

## Abstract

Gastric cancer, one of the most common malignancies worldwide, typically has a poor prognosis and poor survival rate. Previous studies have investigated the chemopreventive effect of celecoxib. In the present study, the SGC-7901 human gastric cancer cell line was utilized to examine the chemopreventive mechanisms of celecoxib. The inhibition of cell proliferation was determined using MTT assay, cell apoptosis was monitored by terminal deoxynucleotidyl transferase-mediated dUTP nick end-labeling (TUNEL) and flow cytometry, and cell ultrastructural changes were assessed via transmission electron microscopy. The mRNA expression of Akt, caspase-8 and -9 was examined using quantitative reverse-transcription-polymerase chain reaction (qRT-PCR) and p-Akt, procaspase-8 and -9 were analyzed via western blotting. The results showed that celecoxib inhibited the proliferation of SGC-7901 cells in a time- and dose-dependent manner. Additionally, celecoxib induced apoptosis as substantiated by typical apoptotic bodies, autophagosomes and an increased apoptotic rate. It was found that following celecoxib treatment, Akt mRNA expression was not significantly altered, and that p-Akt protein levels decreased in a time- and dose-dependent manner. Additionally, caspase-8 and -9 mRNA expression was significantly increased, while procaspase-8 and -9 protein expression decreased relative to the time- and dose-dependent effects. These results demonstrated that celecoxib induced apoptosis and autophagy of gastric cancer cells *in vitro* through the PI3K/Akt signaling pathway. Moreover, our findings suggested that celecoxib induces apoptosis in gastric cancer cells through the mitochondrial and death receptor pathways, providing additional understanding regarding the chemopreventive behaviors of celecoxib and its uses in cancer therapy.

## Introduction

Gastric cancer is among the most common malignancies worldwide, with a 5-year survival rate of only 20% (1). Due to the fact that this type of cancer has a poor prognosis, attention has been drawn to the chemopreventive effect of celecoxib, a cyclooxygenase-2 (COX-2) inhibitor. COX, also known as prostaglandin synthase peroxidase, is the rate-limiting enzyme catalyzing arachidonic acid into prostaglandins, with COX-2 being involved in inflammatory diseases and certain types of tumor (2,3). *In vitro* and *in vivo* studies have shown that the long-term and widespread use of non-steroidal anti-inflammatory drugs (NSAIDs) may contribute to the maintenance of gastric health, with epidemiological studies showing possible risk reduction (4,5).

Apoptosis, programmed cell death (PCD), occurs in multicellular organisms, with autophagy also triggering PCD through different apoptotic mechanisms (6,7). Celecoxib and related compounds have been shown to induce cell cycle arrest, inhibit tumor growth and suppress tumor angiogenesis, with celecoxib potently inducing apoptosis in tumor cells (8–11). An increased expression of COX-2 and Akt in gastric carcinomas relative to normal gastric mucosa, with celecoxib treatment inducing tumor apoptosis has also been shown (12). Further experimentation showed that COX-2 inhibitors may induce apoptosis by affecting Akt phosphorylation, thus activating the Akt signaling pathway (13). In this study, we used the selective COX-2 inhibitor celecoxib to treat the SGC-7901 gastric cancer cells and to induce cell apoptosis *in vitro*. The effects of celecoxib on apoptotic and autophagic cell death through the monitoring of mRNA and protein levels of Akt, caspase-8 and -9 were also examined. This approach aids in further characterization of the apoptotic effect of celecoxib via the PI3K/Akt signaling pathway in order to gain a better understanding of its antitumor effects.

## Materials and methods

### Cell culture

SGC-7901 human gastric cancer cells (Type Culture Collection Committee, Chinese Academy of Sciences, Shanghai, China) were cultured in RPMI-1640 medium (Gibco, Long Island, NY, USA) with 2 mmol/l glutamine, supplemented with 10% fetal bovine serum (FBS; HyClone, Logan, UT, USA), 50 U/ml penicillin, and 50 mg/ml streptomycin. The cells were plated at a density of 1×10^5^ cells/ml in 6-well tissue culture plates and grown to confluency at 37°C with 5% CO_2_. When 50% confluency was reached, serum-supplemented medium was replaced with the recommended serum-free RPMI-1640 medium for overnight culturing before celecoxib intervention. Celecoxib, provided by the Faculty of Medicine, the Chinese University of Hong Kong, stock solution was added to the serum-supplemented medium at different concentrations and cultured until the detection time.

### MTT assay

The inhibition of cell proliferation in SGC-7901 cells following celecoxib treatment was evaluated using an MTT assay (Sigma-Aldrich, Shanghai, China) as per the manufacturer’s instructions. Briefly, 5×10^3^ cells/well were seeded in 96-well plates, incubated in culture medium for 24 h, and treated with varying concentrations of celecoxib (0, 50, 75, 100 and 125 μmol/l) for 24, 48 and 72 h, with parallel samples treated with DMSO only serving as controls. Following treatment, the formation of formazan crystals was measured after 4 h of MTT incubation (10% v/v) at an optical density (OD) of 490 nm, with each experiment repeated in triplicate. The relative cell proliferation inhibition rate was calculated as: (1−OD490_Test_/OD490_Control_) ×100% to show a percentage value.

### TUNEL assay

DNA breaks occur late in the apoptotic pathway and can be determined and analyzed by performing the TUNEL assay (Roche, Basel, Switzerland). Firstly, cells were seeded on coverslips and treated with 100 μmol/l celecoxib for 72 h. Following treatment, the cells were washed, fixed and stained as per the manufacturer’s instructions and apoptotic numbers evaluated using a confocal laser scanning microscope (Leica, Wetzlar, Germany) at 515–565 nm.

### Flow cytometric (FCM) analysis of apoptosis

Apoptosis was assessed by flow cytometric analysis using the Annexin V-FITC/PI apoptosis detection kit (Invitrogen, Life Technologies Ltd., Carlsbad, CA, USA). SGC-7901 cells were seeded in 6-well plates at ~5×10^4^ cells/well. Following treatment with celecoxib, the cells were trypsinized, centrifuged to remove the supernatant, washed with phosphate-buffered saline (PBS), suspended in 100 μl of 1X binding buffer (10 mM HEPES, 140 mM NaCl, and 2.5 mM CaCl_2_), and stained with Annexin V and PI as per the manufacturer’s instructions. FITC-positive and PI-negative cells were considered apoptotic cells, PI-positive cells were considered necrotic, and unstained cells were considered normal viable cells. The apoptotic rates of the various cell groups were calculated and comparisons of apoptotic rates were conducted among the various groups.

### Transmission electron microscope (TEM) analysis of cell ultrastructure

Cells were seeded on coverslips, treated with 125 μmol/l celecoxib for 72 h (with a parallel untreated control), cultured in RPMI-1640 for 72 h, collected and fixed with 3% glutaraldehyde. The cells were washed with PBS, fixed in 1% osmium tetroxide, dehydrated by graded ethanol and acetone, and routinely embedded and polymerized. The slices were contrasted with an aqueous solution of uranyl acetate and lead citrate and examined by JEM-1230 transmission electron microscope (Jeol Ltd., Tokyo, Japan).

### Quantitative reverse-transcription-polymerase chain reaction (qRT-PCR) analysis

SGC-7901 cells were cultured at a density of 1×10^5^ cells/ml in 6-well tissue culture plates. One group was treated with various concentrations of celecoxib (0, 75, 100 and 125 μmol/l) and cultured for 72 h, while an additional test group was treated with 125 μmol/l celecoxib for 0, 24, 48 and 72 h. Total RNA was extracted by a column RNA extraction kit (Sangon, Shanghai, China) and reverse-transcribed into cDNA at 37°C for 15 min, and 85°C for 5 sec. Diluted cDNA was subjected to qRT-PCR using a SYBR^®^ Premix Ex Taq™ II kit (Takara Bio, Inc., Shiga, Japan) in 25 μl of reaction solution containing 2 μl of cDNA template, 1 μM of each primer, 10 μl of 2X SYBR-Green master mix, and brought to the final volume with RNase-free water. Reactions were performed in triplicate via a PCR thermal cycler (Roter-Gene 3000; Corbett, Sydney, Australia) under the following conditions: pre-denaturation at 95°C for 30 sec, 40 cycles of denaturation at 95°C for 5 sec, and annealing at 62°C for 30 sec. The relative expression was calculated by the 2^−ΔΔCT^ formula. The primer pairs for qRT-PCR are listed in Table I.

### Western blot analysis

Total protein was extracted and protein concentrations established via bicinchoninic acid (BCA) assay. Protein (25 μg) was denatured, separated by SDS-PAGE electrophoresis and transferred to a PVDF membrane. After blocking overnight at 4°C using 5% BSA, the membranes were incubated with primary antibodies (anti-procaspase-8 1:2,500 and procaspase-9 1:2,000; both from Abcam, Cambridge, MA, USA), p-Akt 1:800 (Bioworld, St. Louis Park, MN, USA) for 2 h at room temperature, washed by TBST and incubated with the corresponding horseradish peroxidase (HRP)-conjugated secondary antibody at 1:2,000 dilution for 2 h. Bands were visualized using enhanced chemiluminescence (ECL; Applygen, Beijing, China) detection reagents and scanned images were quantified using ImageJ software. Experiments were performed in triplicate with β-actin used as a housekeeping control for normalization. The ratio of target gene to β-actin was used for semi-quantification and comparison between different groups.

### Statistical analysis

Triplicate data are presented as mean values and shown as the means ± standard deviation (SD). Samples were analyzed by one-way ANOVA, with P<0.05 considered to indicate statistical significance.

## Results

### Celecoxib inhibits proliferation of SGC-7901 cells

**Figure 1:**
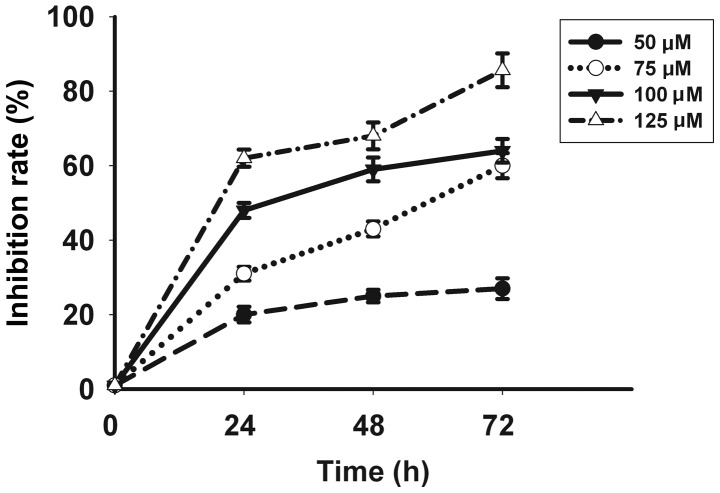
Inhibitory effects of celecoxib on cell proliferation of the SGC-7901 gastric cancer cell line detected by MTT analysis. Following treatment with celecoxib at the indicated concentrations and time-points, SGC-7901 cells showed a significant inhibition of cell proliferation in a time- and dose-dependent manner.

Following *in vitro* treatment with celecoxib, the SGC-7901 gastric cancer cell line showed a significant inhibition of cell proliferation in a time- and dose-dependent manner, with the most pronounced effect evident at a concentration of 125 μmol/l for 72 h as identified by a proliferation inhibition rate of 85.6±4.51% (Fig. 1).

### Celecoxib induces apoptosis of SGC-7901 cells

**Figure 2:**
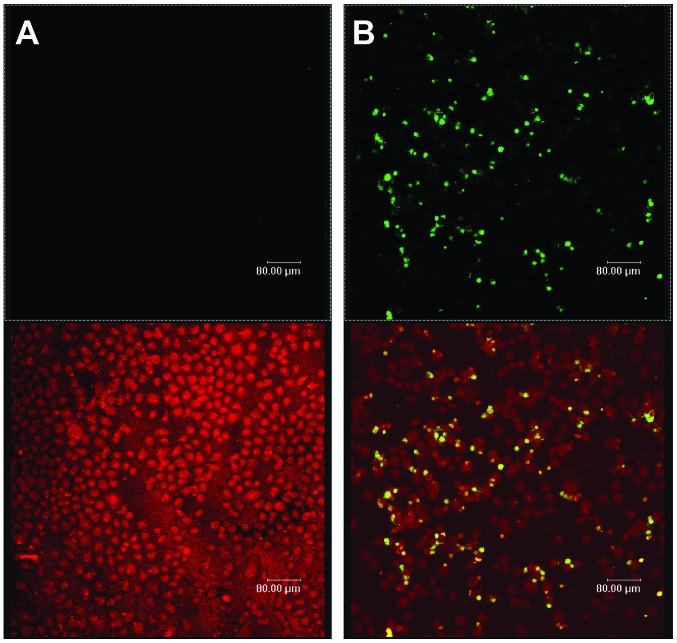
Apoptosis was assessed by terminal deoxynucleotidyl transferase-mediated dUTP nick end-labeling (TUNEL) analysis for SGC-7901 human gastric cancer cells. (A) Control group; (B) group treated with 100 μmol/l celecoxib for 72 h. The apoptotic cell number of the group treated with 100 μmol/l celecoxib for 72 h increased significantly relative to the control. Apoptotic cells with green fluorescence were detected by laser scanning confocal microscopy at an excitation of 515–565 nm, while all the cells exhibit a red image under bright field microscopy. The two images were superimposed to show the specificity of apoptotic cells (yellow) and their position..

Fluorescein- labeled dUTP was connected to DNA 3′-OH ends of apoptotic cells by the deoxynucleotidyl transferase enzyme. Apoptotic cells with green fluorescence were detected by laser scanning confocal microscopy at an excitation of 515–565 nm, while all cells were exhibited as red under bright field microscopy. The two images were superimposed to show the specificity of apoptotic cells (yellow) and their position. Celecoxib-treated cells (Fig. 2B) showed significant levels of apoptosis relative to the control (Fig. 2A), with a statistical significance of P<0.05.

**Figure 3:**
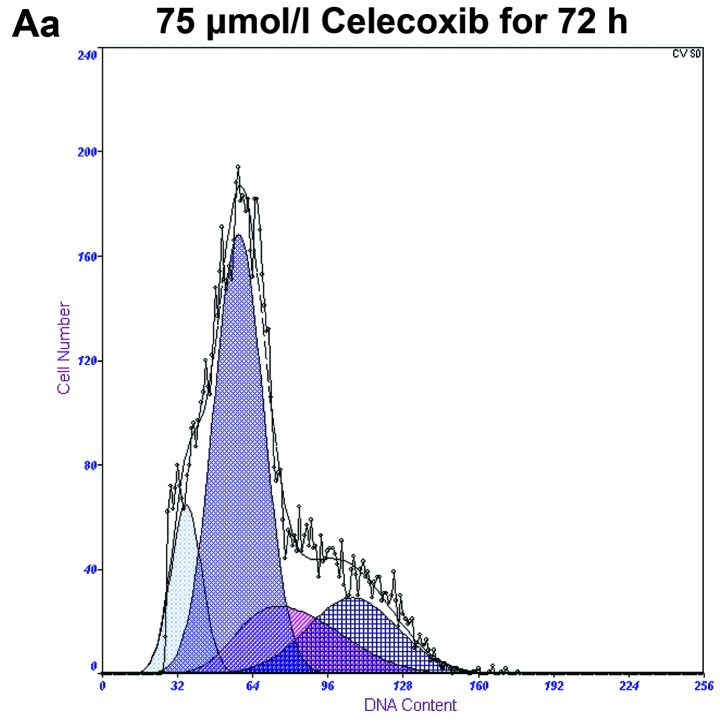

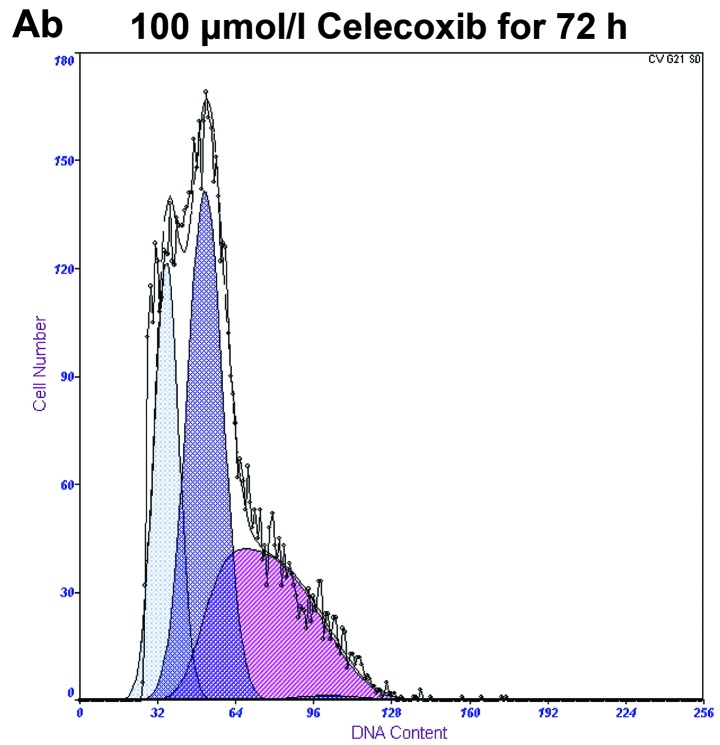

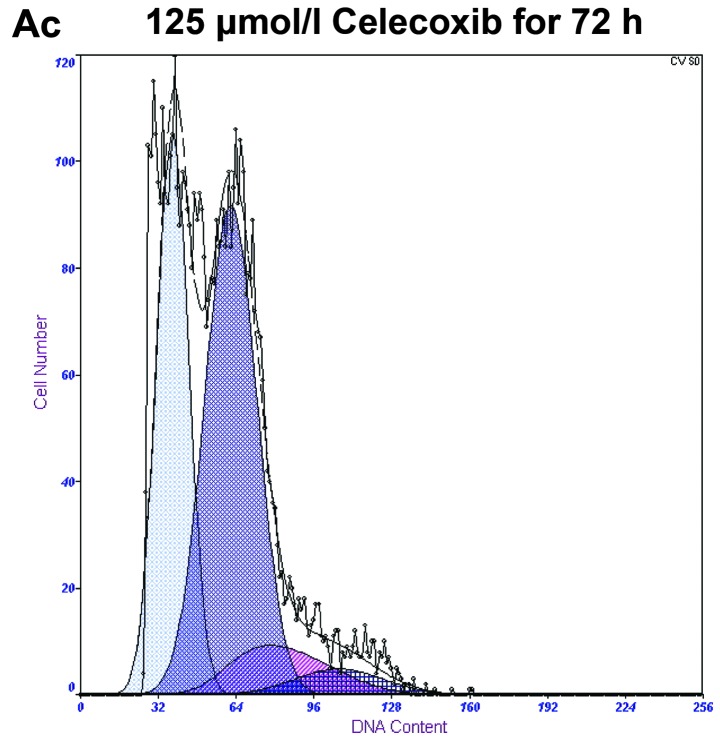

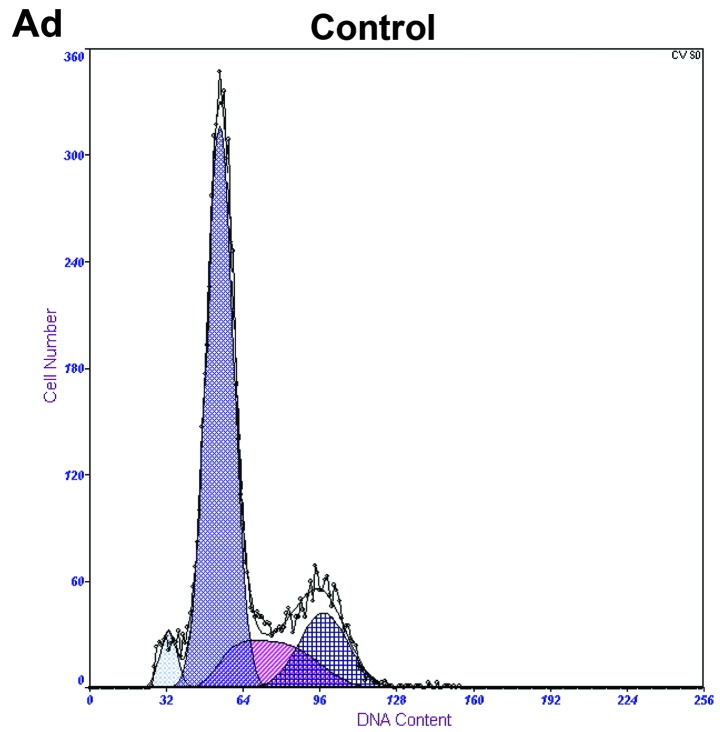

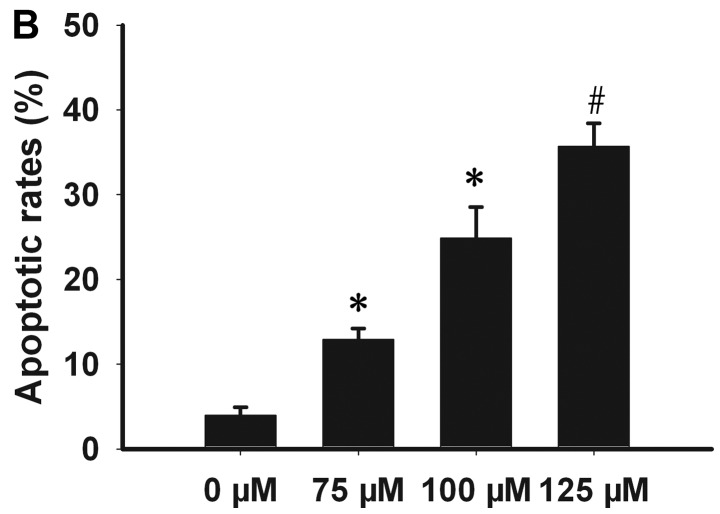

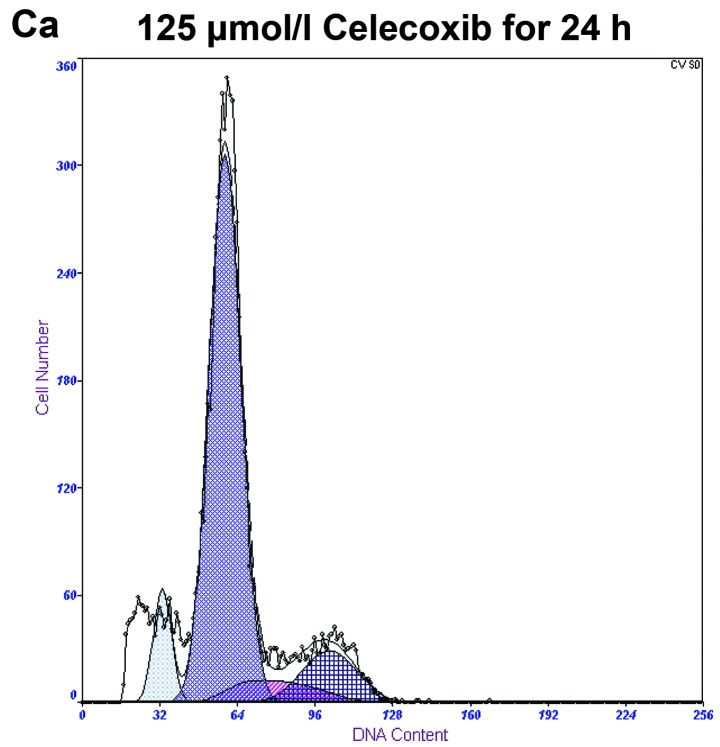

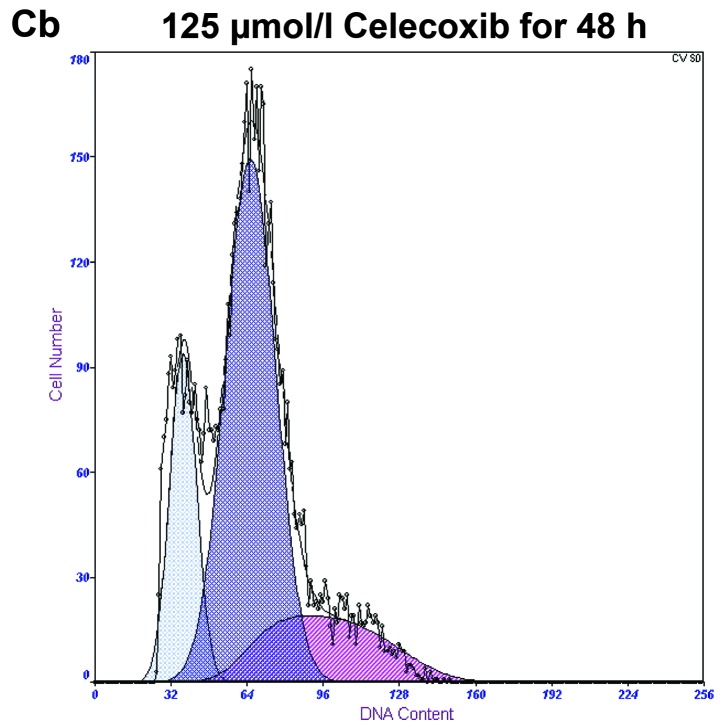

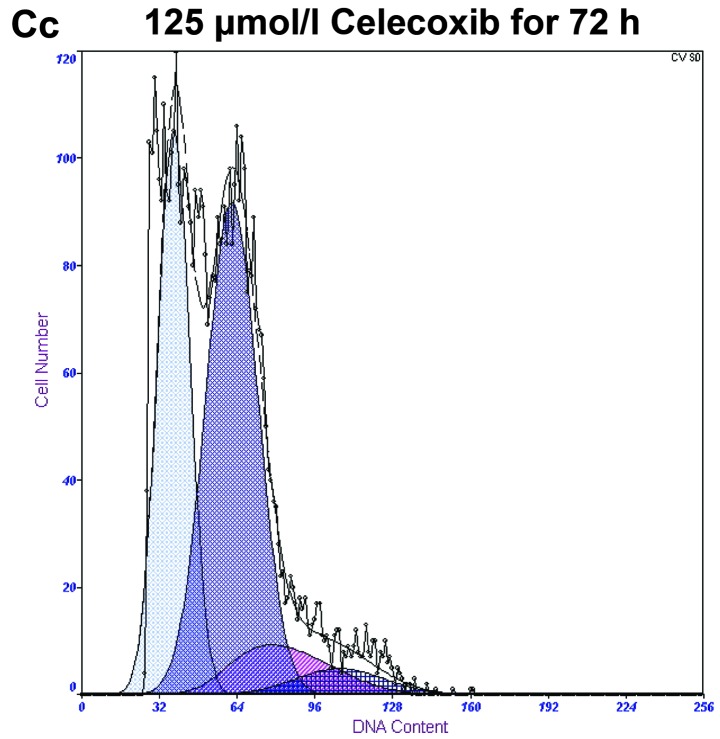

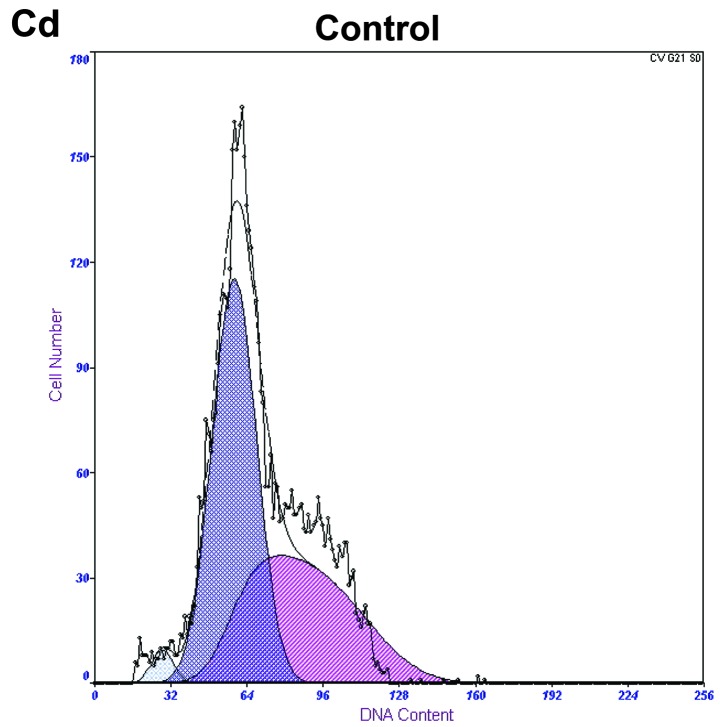

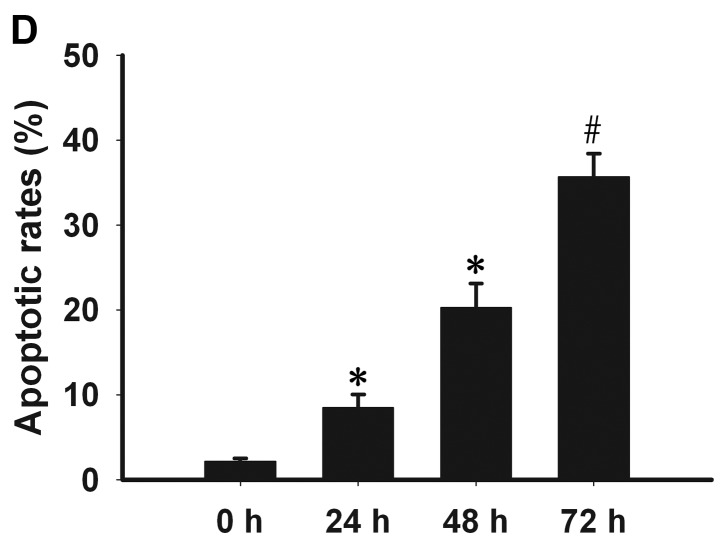
Apoptotic rates of human gastric cancer cell SGC-7901 after treatment with celecoxib at different doses and time-points as detected by flow cytometry. The apoptotic rates of SGC-7901 cells markedly increased in a time- and dose-dependent manner following treatment with 0, 75, 100 and 125 μmol/l of celecoxib for 72 h (A and B). The apoptotic rates of SGC-7901 cells markedly increased in a time- and dose-dependent manner following treatment with 125 μmol/l of celecoxib for 0, 24, 48 and 72 h (C and D). ^*^P<0.05 or ^#^P<0.01 when compared with the control group.

Treatment with 0, 75, 100 and 125 μmol/l of celecoxib for 72 h yielded apoptotic rates of 4.0±0.91, 12.9±1.32, 24.6±3.63 and 35.7±2.73%, respectively, with a statistical significance of P<0.05 when compared to the control group. Treatment with 125 μmol/l of celecoxib for 0, 24, 48 and 72 h, yielded apoptotic rates of 2.2±0.32, 8.5±1.57, 20.3±2.84 and 35.7±2.73%, respectively. Both study sets demonstrated a gradual increase in apoptotic rates in a time-and dose-dependent manner (Fig. 3).

### Celecoxib alters the ultrastructure of SGC-7901 cells

**Figure 4:**
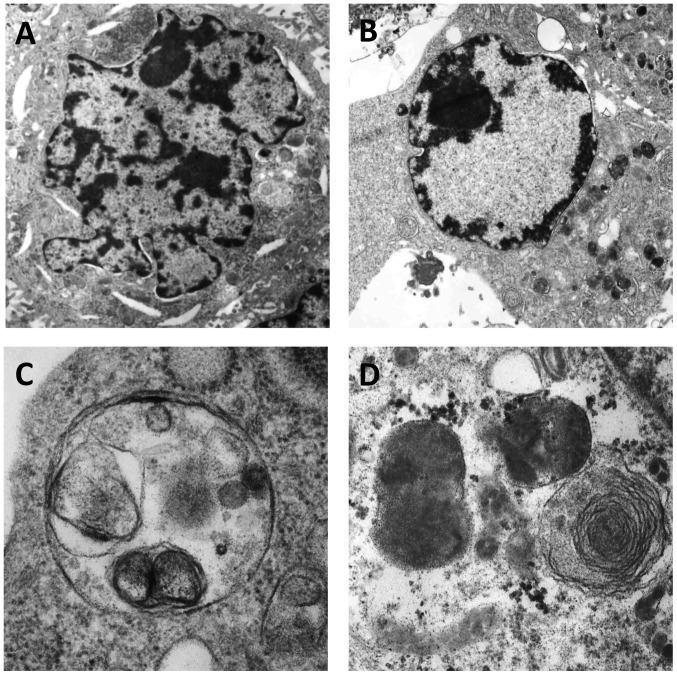
Ultrastructure changes of SGC-7901 cells following treatment with 125 μmol/l celecoxib for 72 h. We observed nuclear membrane shrinkage and retraction in early apoptosis (×5,000) (A), apoptotic body in late apoptosis (×3,000) (B), and changes in autophagy: autophagic vacuolar and autophagosomes (×40,000 or ×30,000) (C and D) by TEM.

Following treatment with 125 μmol/l celecoxib for 72 h, typical early apoptotic changes were found to include nuclear membrane shrinkage and retraction (Fig. 4A), nuclear chromatin condensation, marginalization and crescents, with late apoptotic changes observed by nuclei cleavage into fragments and apoptotic body production (Fig. 4B). Additionally, typical autophagic structures were found to include several cytoplasmic autophagic vacuoles and autophagosomes, which swallowed organelles (Fig. 4C and D).

### Effect of celecoxib on Akt, caspase-8 and -9 expression

**Figure 5:**
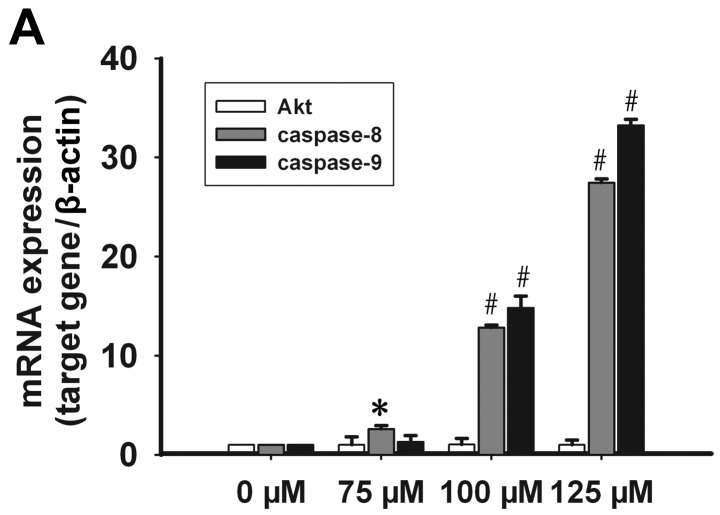

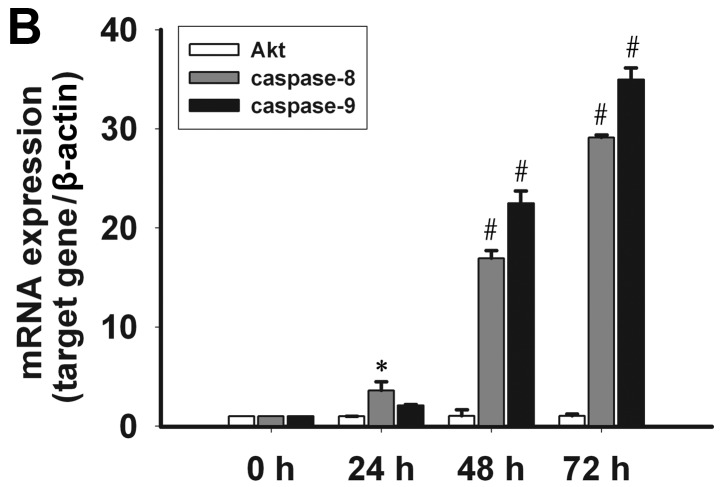
Akt, caspase-8 and -9 mRNA expression of SGC-7901 human gastric cancer cells following treatment with celecoxib at different doses and time-points with the first group including: the control, 75, 100 and 125 μmol/l celecoxib for 72 h (A) and group two including: the control, 125 μmol/l celecoxib for 24, 48 and 72 h (B). ^*^P<0.05 or ^#^P<0.01 relative to the control group from three independent experiments.

**Figure 6:**
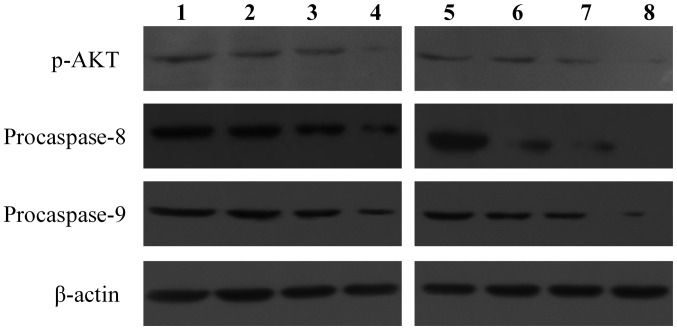
p-Akt, procaspase-8 and -9 protein expression in SGC-7901 human gastric cancer cells following treatment with celecoxib at different doses and time-points. SGC-7901 cells were treated with 0, 75, 100 and 125 μmol/l celecoxib for 72 h (lanes 1–4) and treated with 125 μmol/l celecoxib for 0, 24, 48 and 72 h (lanes 5–8). Procaspase-8, -9 and p-Akt protein expression were significantly lower than the control group in a time- and dose-dependent manner. Aliquots of protein extracts (40 μg) were immunoblotted with the indicated antibodies.

No significant change in the mRNA levels of Akt was observed subsequent to treatment with celecoxib; however, the presence of p-Akt decreased in a time- and dose-dependent manner. Caspase-8 mRNA expression increased in a dose-dependent manner at concentrations of 75, 100 and 125 μmol/l of celecoxib. Caspase-9 mRNA expression levels increased significantly at a concentration of 100 and 125 μmol/l of celecoxib (Fig. 5A). Following treatment with 125 μmol/l of celecoxib for 24, 48 and 72 h, caspase-8 and -9 mRNA expression increased significantly (Fig. 5B). By contrast, procaspase-8 and -9 protein expression was significantly lower than the control group in a time- and dose-dependent manner (Fig. 6). These results showed that celecoxib may inhibit Akt phosphorylation and promote caspase-8, -9 transcription and procaspase-8, -9 protein activation.

## Discussion

Gastric carcinoma is among the most common malignancies worldwide, with an elevated 5-year postoperative mortality rate (14) thus creating a need for an alternative treatment method. Currently, the role of celecoxib, a non-cytotoxic COX-2 inhibitor, in cancer therapy has been under scrutiny (14). COX, also known as prostaglandin synthetase, has three known isoenzymes in mammals, COX-1, COX-2 and COX-3 (15). COX-2 is present at low levels of expression in most normal tissues, but tumor factors, inflammatory cytokines and growth factors could promote its expression (16). COX-2 is related to the development of tumors by promoting tumor cell proliferation, enabling tumor evasion of the host immune surveillance and promoting tumor invasion/metastasis (5,17).

Multicellular organisms maintain their homeostasis through cell proliferation and PCD, with an imbalance possibly leading to the development of cancer. In this experiment, we found that the selective COX-2 inhibitor celecoxib induced apoptosis of SGC-7901 cells via reduced expression levels of COX-2, as obsreved by inhibited cell proliferation using MTT analysis and an increased number of apoptotic cells as detected by TUNEL and flow cytometry. Moreover, typical apoptotic changes were shown to include nuclear membrane shrinkage, nuclear chromatin condensation and apoptotic bodies using TEM to support the apoptotic effects of celecoxib.

Caspases are a type of protease associated with apoptosis and cytokine maturation, and are divided into initiator caspases, effector caspases and inflammatory mediators. Caspases are synthesized as relatively inactive zymogens and must undergo a process of activation during apoptosis. Caspase-8 is the initiator of the Fas-Fas ligand (FasL) pathway, and usually exists in the form of procaspase-8. When FasL binds to the corresponding Fas receptor, the intracellular death effector domain (DED) of the Fas receptor attracts Fas associated with death domain protein (FADD) and recruits procaspase-8 to form a death-inducing signaling complex (DISC). Procaspase-8 is then hydrolyzed to generate activated caspase-8, followed by the activation of procaspase-3 and other effector caspases that eventually induce apoptosis (18). Caspase-9 is the initiator of the mitochondrial pathway, also known as procaspase-9, an inactive zymogen. The initiator caspase-9 is activated by the assembly of a multimeric complex (dubbed apoptosome) involving Apaf-1 and cytochrome *c*. Cleaved caspase-9 and -3 are activated and these effector caspases degrade a large number of cell proteins, ultimately inducing cell apoptosis (19,20). In this study, we found that celecoxib significantly increased caspase-8 and -9 mRNA expression in a time- and dose-dependent manner in SGC-7901 cells, suggesting that celecoxib may activate caspase-8 and -9 to initiated apoptosis through the death receptor and mitochondrial pathways, respectively.

Autophagy is a crucial component of the cellular stress adaptation response that maintains mammalian homeostasis (21). There are three different forms of autophagy that are commonly described: macroautophagy, microautophagy and chaperone-mediated autophagy. Macroautophagy is the predominant pathway occurring mainly to eradicate damaged organelles or unused proteins. Macroautophagy is mediated by a unique organelle, the autophagosome, which encloses long-lived proteins and portions of organelles for delivery to the lysosome (22,23). Autophagy may play different roles in cancer occurrence and progression, while also potentially promoting or inhibiting cell proliferation at different stages of tumor growth (24). For example, autophagy plays a protective role in tumor cells via degradation of organelles under nutritional deficiency. Conversely, autophagy can also inhibit tumor growth via beclin 1, UVRAG, Bif and Atg. Findings of a recent study showed that berberine extracts promoted autophagy by activating beclin 1 expression and activated caspase-9 to induce apoptosis in hepatoma cells (25). Plant lectin from *Polygonatum cyrtonema* induced apoptosis and autophagy by inhibiting the Ras/Raf and PI3K/Akt signaling pathways in murine fibrosarcoma cells (26). In this study, the selective COX-2 inhibitor celecoxib, not only generated morphological changes indicative of apoptosis, but also typical changes of autophagy to include cytoplasmic autophagic vacuoles and autophagosomes.

The molecular mechanism by which celecoxib induces apoptosis is not yet fully understood. The PI3K/Akt pathway widely presents in normal cells, but is abnormally activated in many malignant tumors (27–29). Akt, also known as protein kinase B (PKB), is a central component of the PI3K/Akt pathway, with Akt phosphoregulation impacting a variety of biological activities. In healthy and tumorigenic cells, Akt can be activated in an intracellular manner by hormones, growth factors, and extracellular matrix components (30). Akt regulates cell growth, survival and apoptosis through substrate phosphorylation, with Akt phosphoregulation observed at the Thr308 and Ser473 site, which are both required for activation. Akt is activated as follows: the activated PI3K produces a secondary messenger PIP3 at the plasma membrane, PIP3 then binds an inactive Akt inducing its shift from the cytoplasm to the plasma membrane where Ser124 and Thr450 are phosphorylated, making Akt undergo a conformational change exposing its Thr308 and Ser473 sites. Immediately, phosphoinositide-dependent kinase 1 (PDK1) and phosphoinositide-dependent kinase 2 (PDK2), which are in close proximity to Akt, respectively catalyze the phosphorylation of the exposed Thr308 and Ser473 sites, resulting in the complete activation of Akt. This may trigger a phosphorylation cascade of downstream targets, ultimately impacting the regulation of cell growth and survival, proliferation and apoptosis, angiogenesis, cell migration and numerous biological processes (30–33).

In the present study, following celecoxib treatment in SGC-7901 cells, p-Akt, or activated Akt, was distinctly downregulated, leading to the upregulation of caspase-8 and -9 mRNA expression and increased procaspase-8 and -9 activation. Thus, we hypothesized that celecoxib inhibited the PI3K/Akt pathway by reducing the level of phosphorylation of Akt, which in turn activated the expression and activation of caspase-8 and -9, resulting in apoptosis through the death receptor and mitochondrial pathways in SGC-7901 cells. Notably, we found changes in the cell ultrastructure to include apoptosis and autophagy, suggesting that celecoxib simultaneously induced apoptosis and autophagy, which is consistent with results of previous studies (5,13)). Autophagy is an evolutionarily conserved process that occurs during the growth and development process in many animals, but its specific mechanism of PCD is unclear. Autophagy and apoptosis could coadjust through p53 (35), PI3K/Akt (36) and Bcl-2-beclin 1 (37). Thus, celecoxib may impact both apoptosis and autophagy via the PI3K/Akt signaling pathway in the SGC-7901 gastric cancer cells. The results of this study provide a new theoretical foundation for the antitumor mechanisms of celecoxib and offers new targets for cancer therapy, although these findings should be verified in future investigations.

## Figures and Tables

**Table I:** Primer pairs for qRT-PCR.

Gene name	Accession	Sequence (5′-3′)	Product size (bp)
Caspase-9	NM_001229	F: CCCATATGATCGAGGACATCCA R: ACAACTTTGCTGCTTGCCTGTTAG	186
Caspase-8	NM_001228	F: GGTACATCCAGTCACTTTGCCAGA R: GTTCACTTCAGTCAGGATGGTGAGA	83
Akt	NM_005163	F: GTGGCAGCACGTGTACGAGAA R: GTGATCATCTGGGCCGTGAA	108
β-actin	NM_001101	F: TGGCACCCAGCACAATGAA R: CTAAGTCATAGTCCGCCTAGAAGCA	126
